# Projecting the COVID-19 epidemic risk in France for the summer 2021

**DOI:** 10.1093/jtm/taab129

**Published:** 2021-08-19

**Authors:** Mattia Mazzoli, Eugenio Valdano, Vittoria Colizza

**Affiliations:** INSERM, Sorbonne Université, Pierre Louis Institute of Epidemiology and Public Health, Paris, France; INSERM, Sorbonne Université, Pierre Louis Institute of Epidemiology and Public Health, Paris, France; INSERM, Sorbonne Université, Pierre Louis Institute of Epidemiology and Public Health, Paris, France; Tokyo Tech World Research Hub Initiative, Institute of Innovative Research, Tokyo Institute of Technology, Tokyo, Japan

**Keywords:** Delta variant, spatial heterogeneity, vaccination rollout, immunity, contacts data, crowding

## Abstract

The next weeks will be critical in determining the conditions and timing of the 4th wave of COVID-19 in France. We assessed epidemic risk to assist spatially targeted surveillance and control. Southwest is estimated to be at highest risk, due to summer crowding, low acquired immunity and Delta variant hotspots.

France’s COVID-19 epidemic situation is at a turning point. Case incidence is increasing with the rapid progression of the Delta variant (63% of detected cases carried the L452R mutation as of July 16).^1^^,^^2^ Vaccination rates had been dropping since the end of May,^3^ but recently announced policies have boosted them. Their effect on the pandemic, however, will be inevitably delayed. Incidence, presence of Delta variant, vaccination and infection-acquired immunity are heterogeneous in space, and this may be further exacerbated by summer-season mobility. Here, we propose a risk metric based on five components to identify the departments in mainland France that will be more exposed to sharp surges during summer 2021.

We used hospitalization, COVID-19 incidence, PCR screening and vaccination data from official sources, crowding and contact data from Facebook.^4^ We introduced 5 risk metrics, each highlighting different aspects contributing to the local epidemic risk updated to the last week of June, under the assumption that mobility and crowding will be similar to summer 2020. They are population-level susceptibility ($s$); immunity level among contacts from other departments (${\rho}^{(j)}$); high proportion of Delta variant ($\varDelta$); exposure to Delta variant through cases from other departments (${\varDelta}^{(j)}$) and population crowding during summer season ($c$). These metrics encode the increased risk of sustained local circulation due to low immunity, high exposure to potentially at-risk populations, increased transmission rate due to crowding (SD1). All are defined between 0 (lowest risk) and 1 (highest). Synthesizing these risk metrics into an overall risk indicator for each department, we identified departments at highest risk (Figure 1A–E).

**Figure 1:**
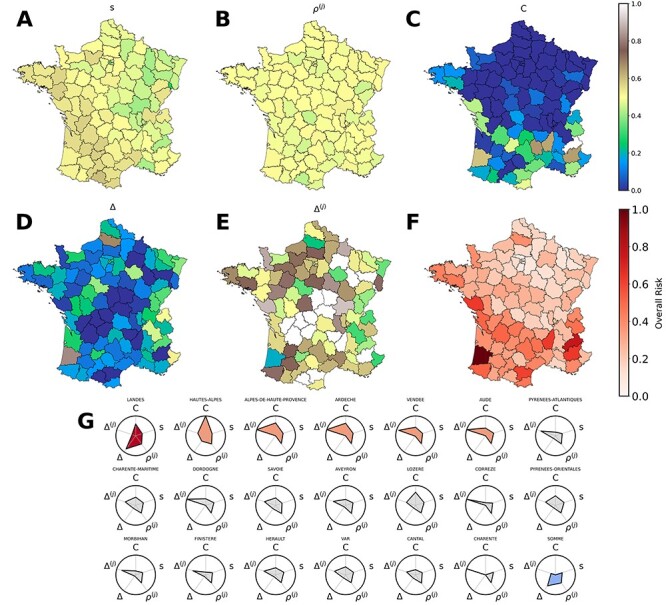
Risk metrics. (A) population-level susceptibility; (B) level of immunity among contacts from other departments; (C) population crowding during summer; (D) proportion of Delta variant among screened cases; (E) exposure to Delta variant through cases from other departments. For the rigorous definition of these risk metrics. see SD1. (F) Overall risk in each department, defined as the mean of the standardized values of the five risk metrics. Values are scaled between 0 and 1. (G) Composition of risk in the top 20 departments by overall risk. Radar plots are colored according to five intervals of overall risk, from 0-to-0.2 (dark blue, low risk) to 0.8-to-1 (red, high risk).

Susceptibility showed a spatial gradient SW (low)—NE (high). This is mostly due to infection-acquired immunity (SD1, Supplementary Figure S1). Crowding was high in the South, low in the North, signaling a net Southward population flow during summer, to coastal and mountain regions. Risk metrics related to the Delta variant were patchy, with no visible spatial trend. This may be due to importations occurring at different times in different places, with long-range mobility providing mixing opportunities between spatially distant departments.

Overall risk is highest in the S-SW (Figure 1F), mainly due to low immunity, summer crowding and early Delta hotspots. Departments at high overall risk exhibit diverse risk profiles (Figure 1G): *Landes* ranks 1st and its risk is dominated by the early spread of the Delta variant; Hautes–Alpes’s risk (2nd) is dominated by high crowding during summer; *Ardèche*’s risk (3rd) is dominated by possible exposure to Delta variant through mobility. Other high-risk departments (e.g. *Lozère*) combine multiple types of risk (crowding, susceptibility, exposure to Delta through contacts).

Our ranking of departments by overall risk is robust across different vaccination scenarios for the following months (SD2, Supplementary Figure S2). We do not consider age structure: our previous work showed little mobility difference across age classes during restrictions.^5^ Younger population strata may be however more mobile and more susceptible during summer holidays. Our assessment does not focus on the impact on healthcare.

Our spatial risk profiling can help inform the prioritization of surveillance and control efforts in the short term.

## Funding

The study was partially supported by Agence Nationale de la Recherche projects DATAREDUX (ANR-19-CE46–0008-03) and EVALCOVID-19 (ANR-20-706 COVI-0007); European Union Horizon 2020 programme grants MOOD (H2020–874850) and RECOVER (H2020–101003589); the EMERGEN project.

**Conflict of interest:** The authors declare no competing interests.

## Authors’ contributions

E.V. and V.C. conceived of and designed the study. M.M. analysed the data and performed the analysis. E.V., M.M. and V.C. interpreted the results. E.V. wrote first draft of the article. E.V., M.M. and V.C. contributed to the critical revision of the final version of the article.

## Supplementary Material

epirisk_summer_supp_taab129
