# Supplementary material for: Projecting the COVID-19 epidemic risk in France for the summer 2021
Source: J Travel Med. 2021 Aug 19;28(7):taab129. doi: 10.1093/jtm/taab129 (PMC8499767; doi:10.1093/jtm/taab129)
Supplement: epirisk_summer_supp_taab129 [file epirisk_summer_supp_taab129.pdf]

Supplementary data

SD1 describes the epidemic risk indicators used.

SD1. Description of the indicators

Population-level immunity

Epidemic records on incidence at department level are obtained from Santé Publique France for the 94 departments of mainland France.[1] To account for the systematic underestimation of SARS-CoV-2 infections occurred all along the pandemic period, we reconstructed cumulated incidence in time, by department (η_i), from hospitalizations.[2] We used a 7-day delay between infection and hospitalization, and hospitalization rates from the literature for each variant Alpha, Beta and Gamma.[3,4] Data on the relative proportion of variants come from screening of positive tests in each department, from February 12 2021 to June 6 2021.[5] Vaccination data come from Assurance Maladie.[6] To account for both vaccine-acquired and infection-acquired immunity we define a metric of immunity ρ_i. This contains the proportion of first- and second-dose vaccinations $v_{1,k}$, $v_{2,k}$, for all available vaccines (k), and their efficacy as found in the literature $\varepsilon_{1,k}$, $\varepsilon_{2,k}$.

$$\rho_i = 1 - (1 - v_i)(1 - \eta_i)$$

$$v_i = \Sigma_k \left(v_{1,k,i}\,\varepsilon_{1,k} + v_{2,k,i}\,\varepsilon_{2,k}\right)$$

We build two scenarios of vaccine rollout:

- optimistic scenario: first doses increase by 10% faster than in June; second doses follow 1st doses according to vaccine-specific guidelines.

- realistic scenario: first doses continue at the same pace of June. Second doses as in the optimistic scenario. The next results are showed for the optimistic scenario, unless explicitly stated.

Mobility and crowding data

We use population and co-location data from the Facebook Data For Good Project. Population data track population in French departments during and before the start of the holidays. Colocation data track mixing in time among residents of different departments. A weekly total number of co-locations between users classified as residents of departments i and j is provided.

A co-location event between departments i and j is recorded when two individuals, one from i and one from j, are in the same 600 × 600m² patch for more than 5 minutes during a period of one week. These records account for repeated encounters. The probability K_{ij} of co-location between i and j is then computed as

$$K_{ij} = \frac{1}{2016}\frac{m_{ij}}{n_i n_j}$$

where n_i is the population of i, m_{ij} is the total number of 5-minute contacts registered between the two populations in one week and 2016 is the number of 5-minutes time slots in the time window. The K_{ij} is then to be intended as the probability of having a contact of 5 minutes in an area of 600 × 600m^2 between people of i and j. We assume $p_{ij} = K_{ij}n_j$, when $i \neq j$, to be a reliable measure of average co-locations made by individuals of i with residents of j. When $i = j$, a systematic overestimation occurs in densely populated cities due to the large area of contact tracing with respect to household sizes. In order to account for this bias, when i = j, we discount those contacts that are due to people staying home 24 hours, a_i. The contact probabilities translate into:

$$p_{ij} = K_{ij}n_j \quad i \neq j$$

$$p_{ij} = (K_{ii}n_i - \frac{n_i}{n_p}a_i) \quad i = j$$

where n_p is the number of co-location patches fitting in the department i area and a_i is the empirical fraction of the total population of i who stay at home 24 hours provided by Facebook Stay Put Data.[7] In this form, p_{ij} represents the average number of contacts with individuals of j registered by an average individual of i.

Summer crowding

Data from summer 2020 show that areas in the Mediterranean coast and the Alps increased their population, while population went down in very densely populated regions like Île-de-France. To assess the difference of population with respect to a baseline situation, we fixed the second week of May 2021 as baseline for our measurements, defining summer crowding as:

$$C_i = \frac{(n_i - n_{i0})}{n_{i0}}$$

Susceptibility

Susceptibility in every department is estimated from vaccinations and estimated cumulative infections (see Supplementary Figure S1). Using the definition of immunity from above, in every department we define the susceptibility risk metric as:

$$s_i = 1 - \rho_i$$

Exposure to lower immunity through contacts with other departments

To account for contacts between different departments with different levels of immunity, we compute the average projected contacts between one department, and all the others, during a given week. We define

$$\rho_i^{(')} = \frac{\sum_j \rho_j p_{ij}}{\sum_j p_{ij}}$$

This metric ranges from 0 (no immunity among contacts) to 1 (full immunity among contacts). From this, we define the metric of exposure to lower immunity through mobility as the relative difference between local and neighbor immunity:

$$\rho_i^{(j)} = \frac{\rho_i - \rho_i^{(')}}{\rho_i + \rho_i^{(')}}$$

Frequency of Delta variant

The presence of Delta variant is now detected through screening of PCR positive tests at department level. Data are provided by Santé Publique France. We use the most recent week of screening data as the most reliable estimation of the proportion of Delta variant among detected cases: Δ_i.

Exposure to Delta variant through cases from other departments

Contacts with individuals from other departments during summer (from mid July to mid August) may expose departments to higher frequency of the variant. Similarly to what we did with immunity, we can compute the exposure to delta infections through contacts with individuals

from other departments, accounting for current cases and current estimations of delta frequency, as:

$$\Delta_i^{(\cdot)} = \frac{\Sigma x_{j,\Delta} p_{ij}}{\Sigma x_j p_{ij}}$$

We compute the risk of exposure to higher frequency of delta through mobility as:

$$\Delta_i^{(j)} = \frac{\Delta_i^{(\cdot)} - \Delta_i}{\Delta_i^{(\cdot)} + \Delta_i}$$

Overall risk

We combine together the five metrics to assess a score of overall risk for each department. We standardize each metric, compute their arithmetic mean, and scale it in the range between 0 and 1.

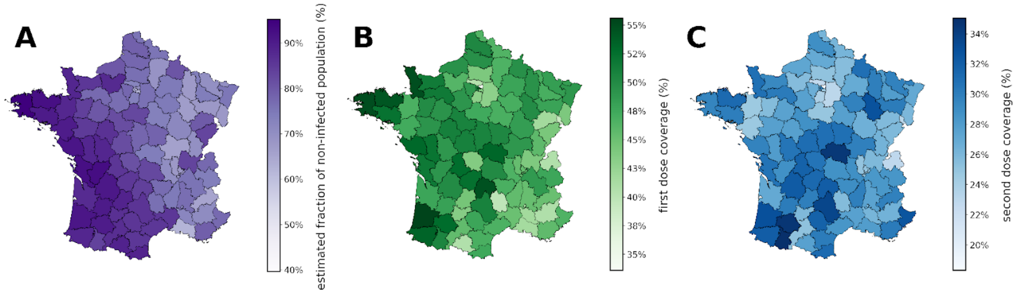

Figure S1. Sources of immunity. A) Current estimated fraction of non-infected population. B) Current 1-dose vaccine coverage; C) Fraction of fully vaccinated individuals.

SD2. Comparing scenarios of projected immunity

We tested two immunity scenarios. S1 includes infection-acquired immunity, complete vaccinations to date, 1st-dose vaccinations to date, projected 1st doses assuming that vaccination rate in each department increases by 10% with respect to June, projected complete vaccinations from recorded 1st doses. S2 includes infection-acquired immunity, complete vaccinations to date, 1st-dose vaccinations to date, projected 1st doses assuming that vaccination rate in each department is the same as the one recorded in June, projected complete vaccinations from recorded and projected 1st doses. Our ranking of departments by

overall risk is robust across the two immunity scenarios (S1 vs S2) of projected vaccination rates in the following months (see Supplementary Figure S2). Pearson coefficient is 0.91, Kendall-tau coefficient is 0.68. This suggests that current heterogeneity in vaccination rollout and assumed rhythms of vaccination for the upcoming weeks are not large enough to affect the ranking in the department risk.

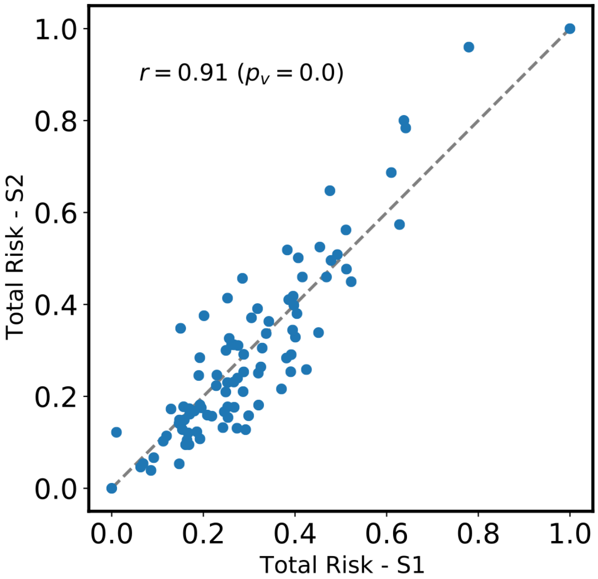

Figure S2. Comparing immunity scenarios. Comparison of the overall risk in the two immunity scenarios (S1 vs S2). Pearson coefficient is 0.91, Kendall-tau coefficient is 0.68.

Supplementary References

[1] Incidence time series at Data Gouv. www.data.gouv.fr/en/datasets/donnees-relatives-aux-resultats-des-tests-virologiques-covid-19/ Accessed: 2021-06-14.

[2] Hospitalized time series at Data Gouv. www.data.gouv.fr/fr/datasets/donnees-hospitalieres-relatives-a-lepidemie-de-covid-19/. Accessed: 2021-06-22.

[3] Funk, T., Pharris, A., Spiteri, G. et al. Characteristics of sars-cov-2 variants of concern b. 1.1. 7, b. 1.351 or p. 1: data from seven eu/eea countries, weeks 38/2020 to 10/2021. Eurosurveillance 26, 2100348 (2021).

[4] Lapidus, N., Paireau, J., Levy-Bruhl, D. et al. Do not neglect SARS-CoV-2 hospitalization and fatality risks in the middle-aged adult population. Infectious diseases now (2021).
[5] Variants screening data.
https://www.data.gouv.fr/en/datasets/donnees-de-laboratoires-pour-le-depistage-indicateurs-sur-les-variants/. Accessed: 2021-06-14.
[6] Vaccination time series from Assurance Maladie France.
https://datavaccin-covid.ameli.fr/explore/dataset/donnees-vaccination-par-tranche-dage-type-de-vaccin-et-departement/information/. Accessed: 2021-06-28.
[7] Facebook Stay Put Data. https://data.humdata.org/dataset/movement-range-maps.
Accessed: 2021-06-18.
